# Chromatin modified protein 4C (CHMP4C) facilitates the malignant development of cervical cancer cells

**DOI:** 10.1002/2211-5463.12880

**Published:** 2020-05-30

**Authors:** Shu‐Li Lin, Mei Wang, Qing‐Qing Cao, Qing Li

**Affiliations:** ^1^ Department of Obstetrics and Gynecology People’s Hospital of Mengyin County China; ^2^ Department of Obstetrics and Gynecology People’s Hospital of Boxing County China; ^3^ Department of Nuclear Medicine The Second Hospital of Shandong University Jinan China; ^4^ Department of Obstetrics and Gynecology Jinan Eighth People’s Hospital Shandong China

**Keywords:** cervical cancer, chromatin modified protein 4C, EMT, proliferation

## Abstract

Despite improvements in prevention and treatment, cervical cancer (CC) still poses a serious threat to women’s health. CHMP4C (chromatin modified protein 4C) is a subunit of the endosomal sorting complex required for transport, which is expressed in both nucleus and cytoplasm. Here, we examined the effect of CHMP4C on the biological behavior of CC cells and the underlying mechanisms. We report that CHMP4C expression is higher in CC tissues, and high CHMP4C expression is associated with lower survival. Up‐regulation of CHMP4C in C‐33A cells accelerates cell proliferation, migration and invasion, whereas down‐regulation of CHMP4C in Ca Ski cells had the opposite effect. Moreover, overexpression of CHMP4C induced activation of the epithelial–mesenchymal transition pathway, whereas depletion of CHMP4C inhibited activation. Our results suggest that CHMP4C contributes to the viability and motility of CC cells by modulating epithelial–mesenchymal transition and may facilitate the identification of novel biomarkers for CC therapy.

## Graphical abstract

Chromatin modified protein 4C is highly expressed in cervical cancer (CC) tissues and cell lines, and contributes to the proliferation, invasion and migration of CC cells by modulating the epithelial–mesenchymal transition. Our findings may facilitate the identification of novel biomarkers for CC therapy.

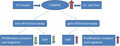

**Abbreviations**
CC: cervical cancer
CCK‐8: Cell Counting Kit‐8
CHMP4C: chromatin modified protein 4C
EMT: epithelial–mesenchymal transition
HPV: human papillomavirus
qRT‐PCR: quantitative real‐time PCR
SD: standard deviation

Cervical cancer (CC) is one of the most common gynecological malignancies [1, 2]. Every year, more than 260 000 women die of CC, mostly in developing countries [3]. The difference in incidence between countries is mainly due to the different awareness of effective screening or preventive treatment [3]. To date, it has been clarified that long‐term recurrent human papillomavirus (HPV) infection is the maximum risk factor for CC [4, 5]. Although effective screening and prevention methods are available, CC still poses a serious threat to women’s health because of its expensive prices and the backward concepts [6]. Hence it is of great clinical significance to explore the molecular mechanism of CC for improving its early diagnosis and treatment.

Chromatin modified protein 4C (CHMP4C) belongs to the CHMP (chromatin modified protein) family. It is a subunit of the endosomal sorting complex required for transport, which is expressed in both nucleus and cytoplasm [7, 8]. Endosomal sorting complex required for transport is involved in the cytokinesis of daughter cells [9], and its dependent extracellular vesicles play an important role in many processes, including the etiology and progression of cancer [10]. Sadler *et al*. [11] found that the polymorphism of CHMP4C increased the susceptibility of cancer and may induce tumorigenesis by disrupting the stability of genome. Based on this feature, CHMP4C has been proved to be unbalanced in many cancers, such as human lung cancer [12], ovarian cancer [13], prostate cancer [14], among others. Nevertheless, no studies have investigated whether it contributes to the malignant progression of CC. According to bioinformatics analysis, we found that CHMP4C was also dysregulated in CC, suggesting that regulating the expression of CHMP4C might be a novel strategy to treat CC. Consequently, based on the published literature and the results of bioinformatics analysis, we chose CHMP4C for further research.

This work aimed to verify the relevancy between the expression of CHMP4C and survival rate of patients with CC, and to explore whether the modulation of CHMP4C expression would impact the biological behavior of CC cells, as well as the regulatory pathways, hoping to discover a novel biomarker that can predict the development of CC.

## Materials and methods

### Data collection

The expression data of CC were downloaded from The Cancer Genome Atlas database (https://www.cancer.gov/about‐nci/organization/ccg/research/structural‐genomics/tcga), including tumor group (*n* = 306) and normal group (*n* = 3). These data were used to analyze the differential expression of CHMP4C in CC tissues and the correlation between CHMP4C expression and outcomes of patients with CC.

### Cell culture

Human CC cell lines (HeLa, C‐33A and Ca Ski) and normal control cells (Ect1/E6E7) were obtained from the cell bank of the Typical Culture Preservation Committee of the Chinese Academy of Sciences. Cells were routinely cultured in Roswell Park Memorial Institute‐1640 medium with 10% FBS, 100 U·mL^−1^ penicillin and 0.1 mg·mL^−1^ streptomycin at 37 °C in 5% CO_2_.

### Cell transfection

siRNA sequences (si‐*CHMP4C*#1: 5′‐CCAAGAAATCTCAGAAG‐3′; si‐*CHMP4C*#2: 5′‐GATGGCACACTTTCTAC‐3′ and si‐con: 5′‐CGAACUCACUGGUCUGACC‐3′) were synthesized from Shanghai GenePharma Co., Ltd. (Shanghai, China), which were used for knockdown of CHMP4C. Simultaneously, for overexpression of CHMP4C in CC cell lines, pcDNA3.1‐*CHMP4C* and corresponding control vector were also purchased from Shanghai GenePharma Co., Ltd. Lipofectamine 2000 Reagent (Invitrogen, Carlsbad, CA, USA) was used for transfection, according to the manufacturer’s protocol.

### RNA extraction and quantitative real‐time PCR

The total RNA of cells was extracted using the TRIzol reagent (Thermo, Waltham, MA, USA) based on the manufacturer’s protocol. After reverse transcription to form cDNA, quantitative real‐time PCR (qRT‐PCR) was carried out to detect the expression of CHMP4C using SYBR Green I (Invitrogen), according to the supplier’s standards. The *CHMP4C* primers (forward: 5′‐AGACTGAGGAGATGCTGGGCAA‐3′, reverse: 5′‐TAGTGCCTGTAATGCAGCTCGC‐3′) were obtained from GENEWIZ (Suzhou, China). Glyceraldehyde‐3 phosphate dehydrogenase was used as internal reference with sequences (forward: 5′‐TGTGTCCGTCGTGGATCTGA‐3′, reverse: 5′‐CCTGCTTCACCACCTTCTTGA‐3′), and the expression of CHMP4C was calculated with the 2^−ΔΔCt^ method.

### Western blotting assay

The radioimmunoprecipitation assay lysate (containing protease inhibitor) was used to crack the cells, and M‐PER Mammalian® Protein Extraction Reagent (Thermo Scientific, Waltham, MA, USA) was used to extract the proteins. The protein concentration was detected using bicinchoninic acid kit. About 20 μg protein was added to each well in the vertical electrophoresis tank, and then electrophoresis was performed with 10% SDS/PAGE. The protein on the gel was transferred to poly(vinylidene fluoride) membrane, followed by blocking with 5% defatted milk powder for 1 h. Then the membrane was incubated overnight at 4 °C with primary antibodies as follows: CHMP4C (1 : 2000; Abcam, Cambridge, UK), E‐cadherin (1 : 500; Abcam, Cambridge, UK), N‐cadherin (1 : 1000; Abcam), Vimentin (1 : 1000; Abcam), Snail (1 : 500; Abcam) and glyceraldehyde‐3 phosphate dehydrogenase (1 : 10 000; Abcam). The next day, the membrane was incubated with horseradish peroxidase–conjugated secondary antibody (Santa Cruz Biotechnology, Inc., Santa Cruz, CA, USA) for 1 h at room temperature. After rinsing, electrochemiluminescence developer was added to develop the images. Glyceraldehyde‐3 phosphate dehydrogenase was used as a control to evaluate the relative protein levels.

### Cell Counting Kit‐8 and colony formation assay

For Cell Counting Kit‐8 (CCK‐8) assay, cells were seeded into 96‐well plates (1000 cells per well) and cultured with CO_2_. We assessed the cell viability at 24, 48 and 72 h, following the standard of the CCK‐8 kit (Dojindo Molecular Technologies, Rockville, MD, USA). The absorbance at 450 nm was measured using a microplate reader (Bio‐Rad, Hercules, CA, USA).

The colony formation assay was performed using the methods described in previous studies [15]; 400 cells were seeded into the culture dish and cultured for 1–2 weeks with 5% CO_2_ at 37 °C. When clones were visible to the naked eye, the cells were fixed with 4% paraformaldehyde and dyed with 0.1% crystal violet dye. Ultimately, numbers of colony were counted.

### Transwell assay

Cell invasion was measured by cell‐penetrating matrix gel‐coated membranes. The invasion assay was conducted as described previously [15]. After transfection for 24 h, 1 × 10^5^ cells were added to the upper chamber with serum‐free medium, and 500 mL serum medium used as the chemical attractant was put to the lower chamber. After incubation, the remaining cells in the upper chamber were erased using a cotton swab. The bottom membrane with invaded cells was fixed with 4% paraformaldehyde and dyed with 0.1% crystal violet. Then the randomly selected fields of vision were photographed for counting.

The migration assay was similar to the invasion assay, but it was evaluated by the penetration of cells into the plain membranes.

### Statistical analysis

SPSS22.0 statistical analysis software (IBM, Armonk, NY, USA) was used to analyze the experimental data. All the assays were repeated three times, and data were presented as the mean ± standard deviation (SD). The discrepancy between two groups was compared with Student’s *t*‐test, and one‐way ANOVA and Dunnett posttest were used to compare the multiple groups. The relationship between gene expression and clinical features was assessed using chi‐square test. Kaplan–Meier analysis was used to plot the survival curve, and the difference between groups was measured using log‐rank test. *P* < 0.05 was considered statistically significant.

## Results

### CHMP4C was highly expressed in CC tissues and cell lines

**Fig. 1:**
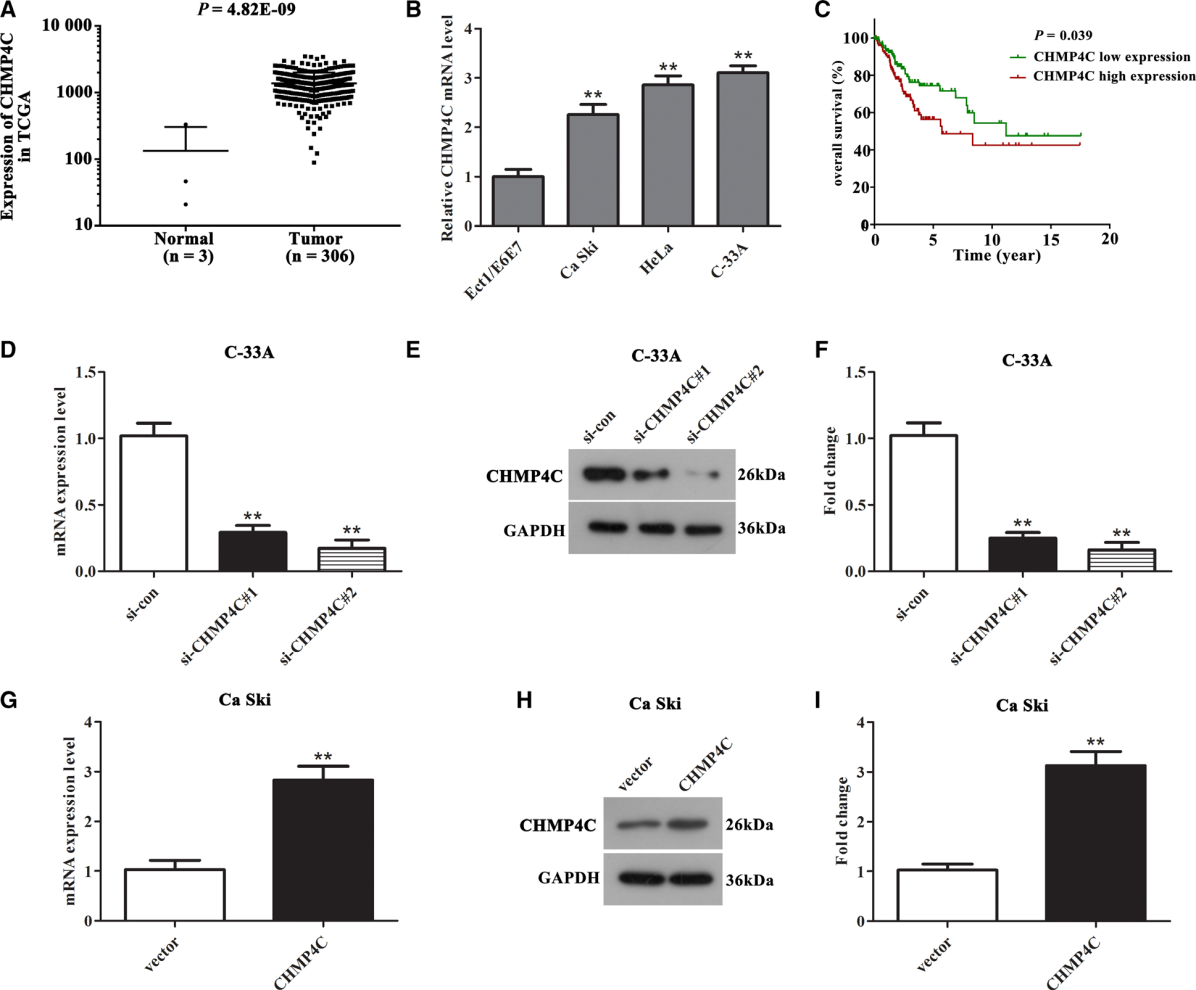
CHMP4C was highly expressed in CC tissues and cell lines, which caused poor outcomes. (A) The expression of CHMP4C in CC tissues was significantly higher than that in healthy control tissues (*P* < 0.001). (B) The mRNA expression level of CHMP4C was up‐regulated in CC cell lines (HeLa, C‐33A and Ca Ski) compared with that in normal control cells Ect1/E6E7. (C) The higher the expression of CHMP4C, the lower the survival rate of patients with CC (*P* < 0.001). (D–F) The CHMP4C expression was obviously declined in C‐33A cells after transfection with si‐CHMP4C#1 and si‐CHMP4C#2 compared with that in the si‐con group. (G–I) The expression of CHMP4C was markedly elevated in Ca Ski cells after transfection with pcDNA3.1‐CHMP4C compared with that in the vector group. All experiments were repeated three times, and the error bars represent SD. **P < 0.01.

CHMP4C has been reported to be imbalanced in many cancers, but whether CHMP4C expression is associated with CC is still unknown. First, the data of control tissues (*n* = 3) and CC tissues (*n* = 306) were downloaded from The Cancer Genome Atlas database to evaluate the differential expression of CHMP4C. It can be seen from Fig. 1A that CHMP4C was highly expressed in CC tissues compared with the control tissues (*P* < 0.01). Subsequently, qRT‐PCR assay was performed to detect the expression of CHMP4C in CC cell lines. The data indicated that the expression of CHMP4C was up‐regulated in different degrees in the CC cell lines (HeLa, C‐33A and Ca Ski) compared with normal Ect1/E6E7 cells (Fig. 1B, *P* < 0.01). Furthermore, C‐33A cells with the highest relative expression were selected for knockdown experiments, whereas Ca Ski cells were chosen for overexpression experiments, which showed relatively low expression.

### High CHMP4C expression caused poor outcomes and was associated with the age of patients with CC

To further analyze whether high CHMP4C expression leads to unoptimistic prognosis, we divided the patients into high‐CHMP4C‐expression and low‐CHMP4C‐expression groups based on the median level, and analyzed the survival rate of the high‐ and low‐expression groups with Kaplan–Meier survival analysis. The results indicated that the higher the CHMP4C expression, the shorter the survival time (Fig. 1C, *P* = 0.039).

Then the relationship between the expression of CHMP4C gene and clinical features was evaluated using chi‐square test. The statistical results in Table 1 demonstrated that the expression of CHMP4C was related to the age of CC patients (*P* = 0.036), and we can clearly observe that patients with age older than 60 years have higher ratio of overexpressed CHMP4C.

**Table 1:** The correlation between CHMP4C expression and clinical features of patients with CC. M, metastasis; N, regional lymph node; T, tumor.

Characteristics	Expression of CHMP4C		*P*‐value
	Low	High	
Age, years			0.036*
<60	127	112	
≥60	25	40	
Grade			0.795
G1 + G2	76	77	
G3	61	58	
Clinical stage			0.515
I + II	119	112	
III + IV	31	35	
Pathologic T			0.150
T1 + T2	107	104	
T3 + T4	11	19	
Pathologic N			0.633
N0	66	67	
N1	32	28	
Pathologic: M			0.615
M0	60	56	
M1	6	4	

^*^ *P* < 0.05.

Moreover, we conducted a Cox regression analysis to investigate whether CHMP4C can be used as an independent predictor for CC prognosis (Table 2). Univariate analysis showed that CHMP4C expression, clinical stage, Pathologic T, Pathologic M and Pathologic N can be used as prognostic factors for CC. Furthermore, multivariate analysis showed that only Pathologic T can be used as an independent prognostic factor for CC.

**Table 2:** Cox regression analysis of CHMP4C as a predictor of prognosis of CC. CI, confidence interval; HR, hazard ratio; M, metastasis; N, regional lymph node; T, tumor.

Variables	Univariate analysis			Multivariate analysis		
	*P*‐value	HR	95% CI	*P*‐value	HR	95% CI
CHMP4C expression（low/high）	0.041*	1.638	1.021–2.628	0.260	1.800	0.647–5.013
Clinical stage（I + II/III + IV）	0.001*	2.286	1.397–3.743	0.215	0.332	0.058–1.895
Pathologic T (T1 + T2/T3 + T4)	0.000*	3.613	1.907–6.846	0.015*	6.442	1.434–28.930
Pathologic M (M0/M1)	0.020*	3.671	1.229–10.962	0.989	0.000	0.000
Pathologic N (N0/N1 + N2 + N3)	0.003*	2.807	1.408–5.593	0.77	2.545	0.904–7.168
Age (<60/≥60 years）	0.054	1.641	0.991–2.715			
Grade (G1 + G2/G3 + G4)	0.681	0.896	0.530–1.513			

* *P* < 0.05.

### Inhibition of CHMP4C in C‐33A cells and overexpression of CHMP4C in Ca Ski cells

To achieve knockdown and overexpression of CHMP4C, we transfected si‐ CHMP4C#1 and si‐CHMP4C#2 into C‐33A cells, respectively, and transfected pcDNA3.1‐CHMP4C into Ca Ski cells. After 24 h, the mRNA and protein levels of CHMP4C in the transfected cells were detected using qRT‐PCR and western blot assay. It can be seen from Fig. 1D–F that transfection with si‐CHMP4C#1 or si‐CHMP4C#2 can obviously reduce the mRNA and protein levels of CHMP4C in C‐33A cells, and si‐CHMP4C#2 showed a higher knockdown efficiency (*P* < 0.01). Meanwhile, as presented in Fig. 1G–I, the CHMP4C expression in Ca Ski cells was markedly increased after transfection with pcDNA3.1‐CHMP4C (*P* < 0.01).

### Knockdown of CHMP4C can inhibit malignant biological behavior of C‐33A cells, whereas overexpression of CHMP4C showed an opposite effect in Ca Ski cells

**Fig. 2:**
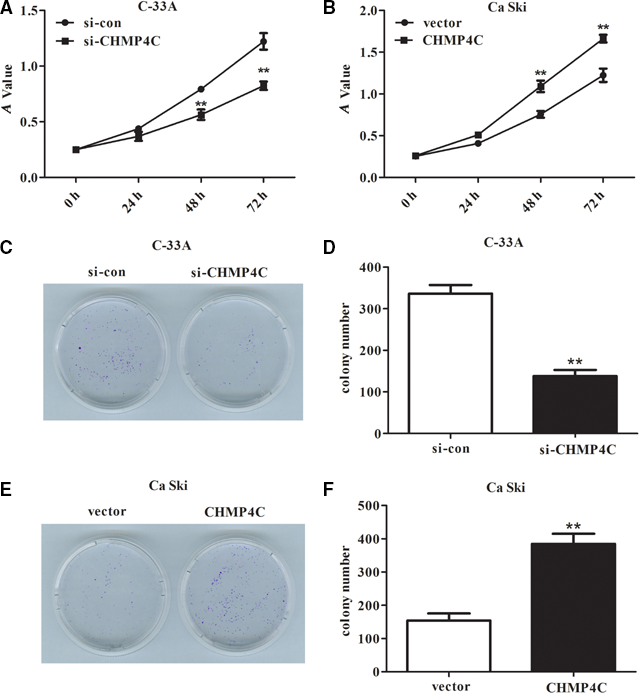
Knockdown of CHMP4C in C‐33A cells inhibited proliferation, and up‐regulation of CHMP4C in Ca Ski cells accelerated proliferation. (A) CCK‐8 assay revealed that deletion of CHMP4C can decrease the cell viability in C‐33A cells. (B) The cell viability was markedly enhanced in Ca Ski cells after overexpression of CHMP4C. (C, D) Colony formation assay revealed that down‐regulation of CHMP4C can reduce the ability of cells to form colonies. (E, F) Up‐regulation of CHMP4C can increase significantly the number of colonies formed compared with that in the vector group. All experiments were repeated three times, and the error bars represent SD. **P < 0.01. *A*, absorbance.

Next, we conducted functional experiments to measure whether CHMP4C contributes to the malignant biological behavior of CC cells. From the CCK‐8 assay, we can see that down‐regulation of CHMP4C can decrease the viability of C‐33A cells compared with control (Fig. 2A, *P* < 0.01), whereas the viability of Ca Ski cells transfected with pcDNA3.1‐CHMP4C was markedly enhanced compared with that transfected with vector (Fig. 2B, *P* < 0.01). The similar results can be seen from the colony formation assay. The colony number was obviously decreased in the si‐CHMP4C group compared with that in the si‐con group (Fig. 2C,D, *P* < 0.01), whereas up‐regulation of CHMP4C in Ca Ski cells can promote the capability to form colonies (Fig. 2E,F, *P* < 0.01).

**Fig. 3:**
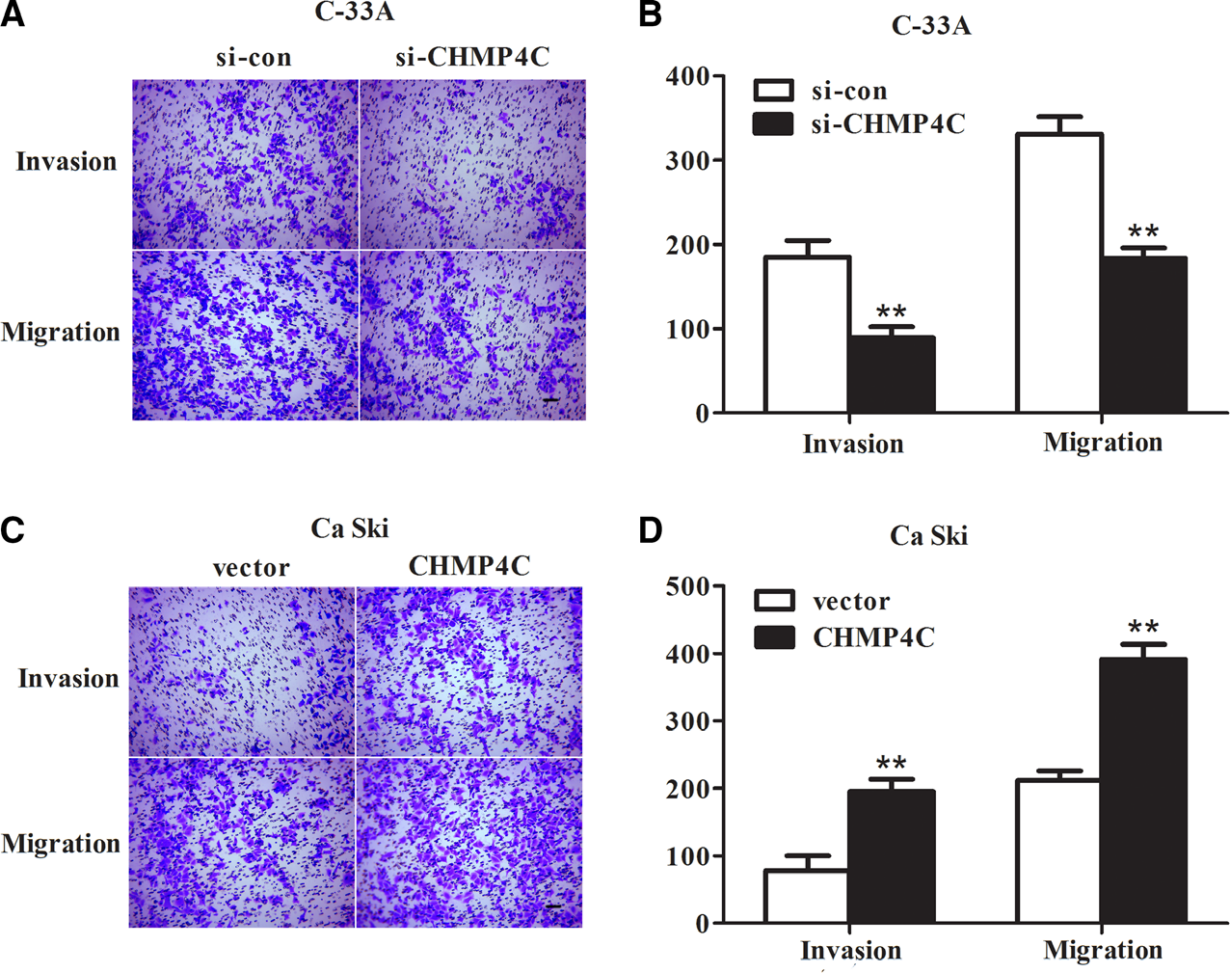
Deletion of CHMP4C suppressed invasion and migration of C‐33A cells, and overexpression of CHMP4C promoted invasion and migration of Ca Ski cells. (A, B) Through the Transwell assay, we found that knockdown of CHMP4C showed a significant inhibition on invasion and migration in C‐33A cells. (C, D) The number of invading and migrating cells was significantly increased after Ca Ski cells transfected with pcDNA3.1‐CHMP4C compared with that in the vector group. Scale bars: 200 μm (A, C). All experiments were repeated three times, and the error bars represent SD. **P < 0.01.

Transwell assay was performed to detect the influence of knockdown or overexpression of CHMP4C on cell migration and invasion. It can be seen from Fig. 3A,B that C‐33A cells transfected with si‐CHMP4C resulted in a marked reduction on the number of invaded and migrated cells compared with the si‐con group (*P* < 0.01), whereas overexpression of CHMP4C in Ca Ski cells showed opposite results (Fig. 3C,D, *P* < 0.01). Taken together, these results illustrated that CHMP4C functioned as an oncogene in the malignant biological behavior of CC cells, thus possibly being involved in the development of CC tumors.

### Epithelial–mesenchymal transition pathway mediates the regulation of CHMP4C on the malignant biological behavior of CC cells

**Fig. 4:**
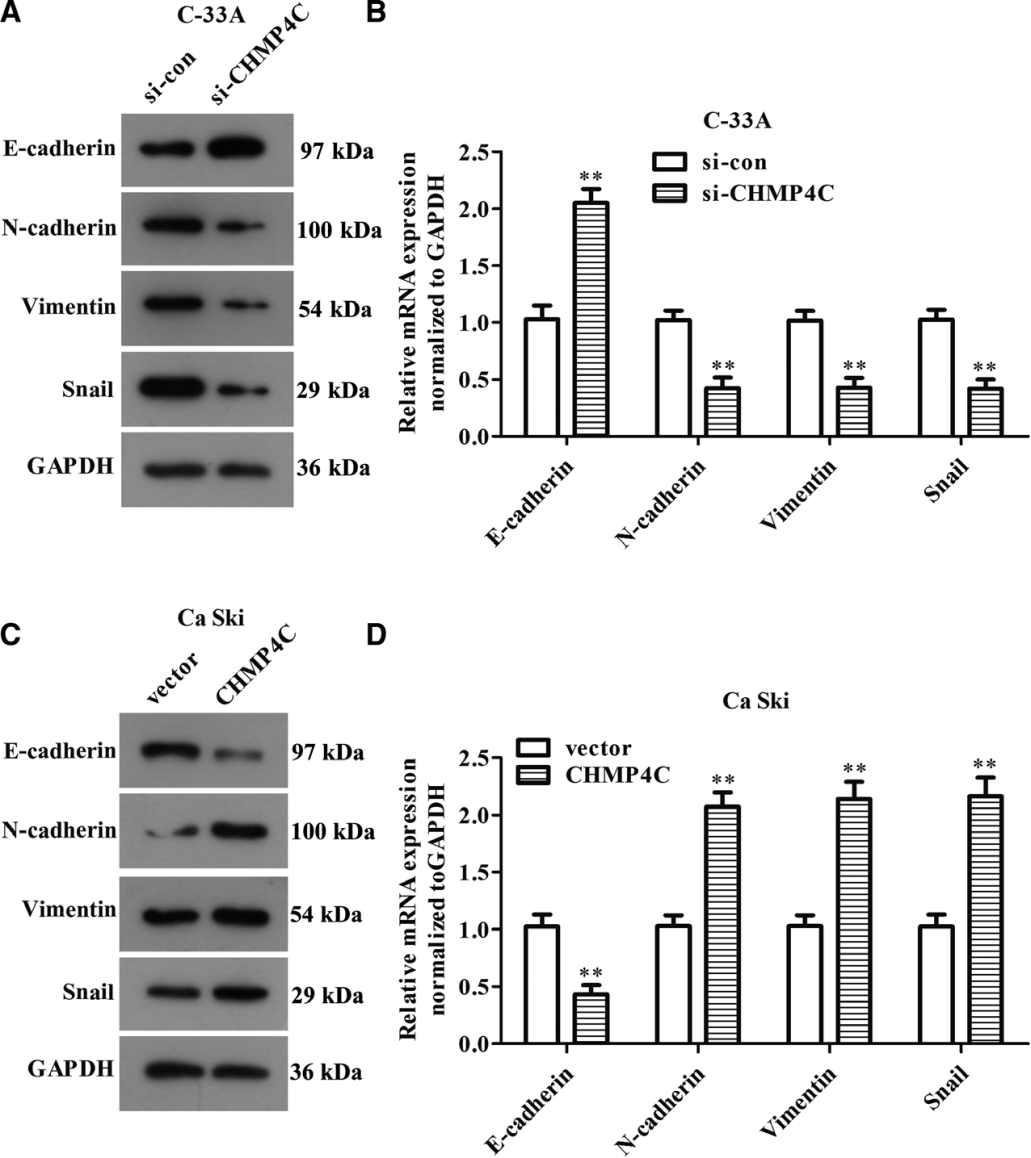
CHMP4C mediates the occurrence of EMT in CC cells. (A, B) Down‐regulation of CHMP4C suppresses the EMT pathway in C‐33A cells. (C, D) Up‐regulation of CHMP4C promotes the EMT pathway in Ca Ski cells. All experiments were repeated three times, and the error bars represent SD. **P < 0.01.

Epithelial–mesenchymal transition (EMT) is a key event that promotes the migration and invasion of resting tumor cells [16]. Thus, we performed western blotting assay to detect the expression of EMT‐related proteins in C‐33A and Ca Ski cells, to assess whether the EMT pathway mediates the regulation of CHMP4C on CC cells malignant phenotype. The results in Fig. 4A,B indicated that knockdown of CHMP4C can increase the protein expression of E‐cadherin, whereas the expressions of N‐cadherin, Vimentin and Snail decline (*P* < 0.01). Meanwhile, overexpression of CHMP4C in Ca Ski cells presented the opposite outcomes (Fig. 4C,D, *P* < 0.01). Because the absence of E‐cadherin is considered to be a marker of EMT, these results illustrated that inhibition of CHMP4C can inhibit the EMT of CC cells. These data prompted a possibility that CHMP4C may contribute to the regulation of CC malignant progression through regulating the EMT pathway.

## Discussion

The occurrence and development of CC is a complex process of multifactor regulation [17]. Currently, the incidence of CC shows a trend of youth [18]. In spite of some progress being made in the potential mechanism of the occurrence and malignant progression of CC, the prognosis of current treatment is still poor [19], so it is an urgent problem to find an effective therapeutic target. In this study, we first found that the expression of CHMP4C was elevated in both CC tissues and cell lines. High CHMP4C expression leads to poor outcomes and is associated with the age of patients with CC. Subsequently, functional experiment indicated that CHMP4C promotes proliferation, migration and invasion of CC cells, in part by activating EMT.

The function of CHMP4C in cell dynamics and endocytogenesis has been widely reported [20, 21, 22]. Based on the role of CHMP4C in a variety of cell functions, research has begun to focus on whether it contributes to the progression of cancer. It has been found that CHMP4C has abnormal expression in cancer. Nikolova *et al*. [23] found for the first time that CHMP4C was highly expressed in ovarian cancer tissues, and its high expression may be related to the poor outcomes of ovarian cancer. It is worth noting that CHMP4C has been identified as a new susceptibility gene for ovarian cancer [24]. In addition, Li *et al*. [12] demonstrated that CHMP4C can promote cell survival and enhance radiation resistance of tumor cells in human non‐small cell lung cancer cells, which functioned as a carcinogenic gene. Most importantly, it has been found that HPV E6/E7 plays an important role in HPV‐induced carcinogenesis [5]. Inhibition of HPV E6/E7 expression results in a significant increase in exosomes, whereas CHMP4C stimulation can promote exosome production and induce its apoptosis [25]. Because HPV infection is a risk factor for CC, we speculated that CHMP4C may also be involved in the pathogenesis and progression of CC. Our data showed that CHMP4C was highly expressed in CC and was associated with poor prognosis, which can be used as a prognostic factor for CC. Further functional experiments indicated that CHMP4C accelerated the malignant biological behavior of CC cells, and it functioned as an oncogene in CC.

EMT is considered to be the key factor to accelerate tumor invasion and migration [16], which can be regulated by many factors, such as miRNA, mRNA, long noncoding RNA [26], etc. Activation of the EMT pathway in tumor cells can promote the development of almost all malignant tumor‐related features [27]. Deletion of E‐cadherin and overexpression of vimentin are hallmarks of EMT activation [28], and miRNA can function in the regulation of CC by regulating E‐cadherin [29]. Some studies have shown that the loss of epithelial features (E‐cadherin, etc.) and the acquisition of mesenchymal properties (N‐cadherin, vimentin, snail, etc.) can improve the invasion of tumors [30]. Notably, high expression of snail is closely related to the deterioration of human cancer [31]. Extensive studies have confirmed that EMT is involved in the malignant progression of many cancers, such as colon cancer [32], ovarian serous cancer [33], breast cancer [34], among others. So far, studies have also reported the association between the progression of CC and EMT. EMT accelerates the invasion and metastasis of primary CC, and plays an important role in the lymph node metastasis of CC [35]. Our findings revealed that up‐regulation of CHMP4C in CC cells can activate the EMT, and thus promote the invasion and migration of CC cells. Knockdown of CHMP4C presented the opposite outcomes. Combined with the data in this article, we concluded that overexpression of CHMP4C induces the occurrence of EMT in CC cells, thereby accelerating cell invasion and migration.

In summary, our study found for the first time that CHMP4C was highly expressed in CC, and its high expression caused poor outcomes. Furthermore, CHMP4C may promote cell proliferation, invasion and migration by activating the EMT pathway. These findings prompted a novel strategy for CC therapy; however, the potential application in clinical treatment still needs further exploration.

## Conflict of interest

The authors declare no conflict of interest.

## Author contributions

S‐LL, MW and QL performed the experiments. S‐LL and MW analyzed the data. S‐LL and Q‐QC wrote the manuscript. S‐LL, Q‐QC and QL reviewed and edited the manuscript. All authors read and approved the final manuscript.
